# Is Social Support a Cause or Consequence of Depression? A Longitudinal Study of Adolescents

**DOI:** 10.3389/fpsyg.2018.01634

**Published:** 2018-09-04

**Authors:** Ping Ren, Xingna Qin, Yunyun Zhang, Ruiping Zhang

**Affiliations:** ^1^Collaborative Innovation Center of Assessment toward Basic Education Quality, Beijing Normal University, Beijing, China; ^2^Department of Education, Zhengzhou University, Zhengzhou, China

**Keywords:** teacher support, peer support, depression, adolescents, longitudinal study

## Abstract

A large body of literature has examined the relations between social support and depression. However, the exact nature and direction of these relations are not well understood. This study explored the relations between specific types of social support (peer support and teacher support) and depression. Adolescents (ages 11 to 17) for the first time (*N* = 2453) participated in a two-wave, 6-month longitudinal study. Structural equation modeling was used to test a social causation model (deficits in social support increase the likelihood of depression), interpersonal theories of depression (depression leads to social erosion), and a reciprocal influence model. Depression influenced peer support significantly and negatively. By contrast, the social causation model was not supported. These results held for males and females. Findings suggested that depression resulted in social support erosion. However, the effect was specific to perceived peer support but not to perceived teacher support.

## Introduction

Adolescence is a time of transition owing to rapid changes in physical, psychological, and social development (Vansteenkiste et al., 2012; Hein et al., 2015). This transitional period is characterized by a decline in parental reliance and an increase in interactions with teachers and peers (Soenens et al., 2012). Depression is the most common psychiatric problem faced by adolescents. A meta-analysis by Costello et al. (2006) reported that the prevalence of depressive disorder in 13- to 18-year-old adolescents was 5.6%. Previous studies have indicated that adolescent depression is associated with future academic failure, poor mental health, suicide, and functional impairment (Sowislo and Orth, 2013).

Numerous studies have explained how people increase and maintain their social support in order to decrease depression. However, does low social support indeed contribute to depression? Many studies suggest that social support is linked to low depression (e.g., Stice et al., 2004; Wight et al., 2006; Ellonen et al., 2008; Dingfelder et al., 2010). Family support (especially parental support) and friend support may reduce adolescent depression (Zhang et al., 2015; van Harmelen et al., 2016; Chang et al., 2018). Teacher support may be also a protective factor against depression (e.g., Reddy et al., 2003; Rueger et al., 2010, 2016). A 5-year longitudinal study indicated that teacher emotional support decreased adolescent depression (Pössel et al., 2013). However, the exact nature and direction of the relation between social support and depression have not ultimately been understood. The arguments about the relations between perceived social support and depression are as follows.

First, social causation model assumes that social support is an antecedent of well-being, and lack of social support causes psychological distress (Kaniasty and Norris, 2008). This model explains the social support-to-distress relationship and predicts that social support mitigates the likelihood of depression (Windle, 1992; Calsyn and Winter, 2002; Needham, 2008; Zhen et al., 2018). Social support may relieve depression through improving self-esteem and decreasing negative cognition (Zang et al., 2017). There are considerable empirical evidences supporting the protective role of social support on adolescent depression (e.g., Windle, 1992; Dingfelder et al., 2010; Rueger et al., 2016; Yu et al., 2016). Reduced levels of perceived support have predicted increased levels of depressive symptoms during adolescence (Windle, 1992). In an 18-month longitudinal study of Chinese middle school students, Yu et al. (2016) reported that teacher support in the fall of 7th grade led to decreased depression in the spring of 8th grade. Studies that investigated both parental support and peer support found that only the former showed prospective effects (Stice et al., 2004; Auerbach et al., 2011), which indicated that specific domains of social support played differential roles with respect to depression.

Additionally, interpersonal accounts of depression suggest that negative self-statements, complaints, repeatedly seeking reassurance, and social inadequacy exhibited by depressed and depression-prone individuals disrupt social relationships (Joiner, 2002; Rudolph et al., 2008). It is theorized that depressed and depression-prone individuals induce negative responses and create interpersonal difficulties in their interactions with others, which cause people to avoid or reject them. In a 2-year longitudinal study, Stice et al. (2004) found that depressive symptoms predicted subsequent reductions in perceived peer social support among female adolescents, but not in perceived support from parents. Similarly, a randomized prevention trial provided experimental support through a 1-year follow-up that depression was negatively associated with perceived social support, but this effect may be confined to perceived friend support rather than parental support for adolescents and was more pronounced for females and for younger adolescents (Stice et al., 2011).

Theorists have also proposed that support and depression may have a reciprocal relationship (Lazarus and Folkman, 1984). People will have depression when they lack of social support, and when depressed, people are less prone to obtain or sustain relationships, thus resulting in a negative spiral (Hansell and Damour, 2005; Cooley et al., 2010). Bidirectional longitudinal associations were found between parental and friendship support and depressive symptoms in Swiss adolescents (Burke et al., 2017). Needham (2008) also indicated that adolescents’ perceived parental support influenced their depression, which in turn influenced their perceived parental support in young adulthood.

Although previous studies have investigated the respective relations between peer support and teacher support and depression, researchers have rarely examined the associations between peer support, teacher support, and depression in the same study. It is of great importance in identifying such an effect to best guide the design of targeted prevention and intervention programs for adolescent emotional problems (Yu et al., 2016). This study aimed to test whether reciprocal relations exist between depression and perceived support, especially from teachers and peers. Previous research has shown that girls are prone to perceive more teacher support and peer support (e.g., Wentzel et al., 2010) and to be more depressed than boys (e.g., Twenge and Nolen-Hoeksema, 2002). Therefore, we also examined whether the relation between social support and depression differed for males and females.

For adolescents, gaining independence and autonomy from parents is a central developmental task (Needham, 2008). Therefore, adolescents’ social relationships with peers and teachers play an increasingly salient role during this stage. Teacher support was shown to become increasingly important in decreasing students’ problem behavior and contributing to classroom adjustment and mental health during adolescence (Colarossi and Eccles, 2003; Wentzel et al., 2010). Peers play a significant role in the lives of young people. Given the importance of peer and teacher relationships to adolescents, this study will consider how peer support and teacher support are correlated with depression.

In the current study, we revealed the relation between social support and depression. The central purpose of this study was to assess the models of low social support and depression by analyzing the available longitudinal data on adolescents. We also examined whether the relation between social support and depression differed by sex in adolescents.

## Materials and Methods

### Sample and Procedure

A sample of 2834 students were randomly selected from 47 classes in grade 7 from seven secondary schools in Mainland China in December 2015. We then tracked 2551 students in May 2016. Students with missing value were excluded from the data analysis due to the low missing rate (3.8%). Thus, a total of 2453 children (50.5% male, 49.5% female) participated in two waves of the study over 6 months and were screened into the final model. The participants ranged in age from 11 to 17 years, and the mean age at Time 1 was 13.02 years (*SD* = 0.61). The children in the sample were all Chinese speaking.

Participation in the study was entirely voluntary and written informed consent was required to conduct the questionnaires at each time wave. All parents or legal caregivers also provided the corresponding written informed consent for the study. Parents’ questionnaires were also administered in our project, which were not used in this study. Under the direction of a study author, students provided data and completed all questionnaires in their classrooms during school hours. Six months later, a second school visit was administered, with attempts made to track all previous participants.

### Measures

#### Children’s Depression Inventory

Children’s depression inventory (CDI; Kovacs, 2003) is a 27-item questionnaire for assessing the cognitive, behavioral, and affective symptoms of depression for children aged 8∼18 years. Each item consists of three statements of varying severity from 0 to 2. Higher scores indicate more depressive symptoms. The CDI was found to have good reliability and validity, as reported in the manual (Kovacs, 2003). Cronbach’s α at Time 1 was 0.87 and Time 2 was 0.88.

#### Perceived School Climate

Perceived school climate is a 25-item version assessing three dimensions of school climate. In this study, two dimensions were chosen: teacher support (e.g., My teachers care about me), which focused on emotional support and included one item related to academic support, and student-student support (e.g., Students help one another). All items are scored on a 4-point rating scale, ranging from (1) “never” to (4) “always.” Higher scores indicate a high level of support in the classroom. Good psychometric properties have been reported in China (Jia et al., 2009). For the current study, each subscale has demonstrated to have acceptable reliability. Cronbach’s α for teacher support (7 items) was 0.79 at Time 1 and 0.81 at Time 2, and for peer support (13 items) 0.86 at Time 1 and 0.85 at Time 2.

### Statistical Analyses

Descriptive statistical analyses and correlation analyses were conducted using SPSS22.0. To examine the theoretical model, Mplus 7.11 was used to estimate structural equation models (SEM). We employed full information maximum likelihood estimation procedure, which is robust against violations of multivariate normality assumptions (Muthén and Muthén, 2010). Model fit was assessed using a Chi-Square (χ^2^), Tucker–Lewis index (TLI), Comparative Fit Index (CFI), and Root Mean Square Error of Approximation (RMSEA). A non-significant χ^2^, TLI and CFI ≥ 0.95, and RMSEA < 0.06 were considered to indicate adequate fit to the data (Kline, 2010; Marshall et al., 2014). A CFI or RMSEA difference between models of <0.01 is considered invariant (Little, 2013). Chi-square difference tests (Δχ^2^) were employed to determine significant differences between theoretical models. Bootstrapping procedure was used to test the significance of the best-fitting model.

## Results

### Preliminary Analyses

Means and standard deviations are given in **Table 1**, and bivariate correlations are displayed in **Table 2**. **Table 1** shows the means and standard deviations. Note that the skewness and kurtosis indices are between −1 and +1, which show that all variables considered in the present study are normally distributed (Aluja et al., 2007). **Table 2** presents the correlations between the key variables. Alpha reliabilities ranged from 0.79 to 0.88, supporting reliability. The variables of interest were significantly related to each other. Teacher support and peer support were positively correlated with each other (*r*s from.35 to.49). Depression was negatively correlated with teacher support and peer support (*r*s from −0.40 to −0.23). Teacher support, peer support, and depression showed highly stability over time (6-month test–retest *r* = 0.54, 0.62, 0.64, respectively). The above findings offered preliminary evidence supporting the hypothesized relations among variables and allowed us to further examine the hypothesized model.

**Table 1:** Means, standard deviations, and reliabilities.

	Mean	*SD*	Skewness	Kurtosis	Reliability
Sex	0.50	0.50	–	–	–
Age	13.00	0.61	–	–	–
**Wave 1**					
Teacher support	2.99	0.53	-0.42	-0.27	0.79
Peer support	3.24	0.49	-0.49	-0.13	0.86
Depression	0.49	0.28	0.73	0.27	0.87
**Wave 2**					
Teacher support	2.79	0.59	-0.31	-0.27	0.81
Peer support	3.16	0.49	-0.40	-0.13	0.85
Depression	0.53	0.30	0.64	0.12	0.88

*Note. Sex is coded so that 1 = boy and 0 = girl. Dashes indicate that the index was not estimated.*

**Table 2:** Bivariate correlations and alpha reliabilities.

Variable	1	2	Wave 1			Wave 2		
			3	4	5	6	7	8
1. Gender	1							
2. Age	-0.06**							
**Wave 1**								
3. Teacher support	0.03	-0.04	1					
4. Peer support	0.14**	-0.07**	0.47**	1				
5. Depression	0.02	0.04	-0.32**	-0.39**	1			
**Wave 2**								
6. Teacher support	-0.01	-0.02	0.54**	0.35**	-.29**	1		
7. Peer support	0.15**	-0.06**	0.36**	0.62**	–	0.49**	1	
8. Depression 2	0.02	0.04*	-0.23**	-0.28**	0.64**	-0.35**	-0.40**	1

*Note. N = 2453. Test–retest correlations appear in bold. ^∗∗^p < 0.01.*

### The Relation of Teachers’ and Peers’ Support to Adolescents’ Depression

**FIGURE 1:**
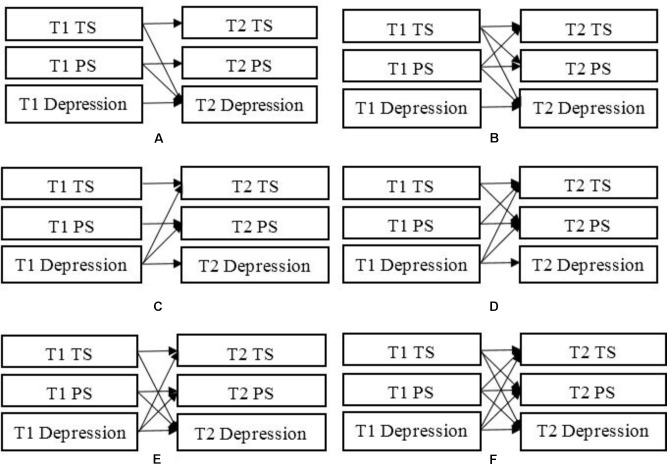
Steps to test the reciprocal effects between teacher support, peer support, and depression. **(A)** tested teacher support and peer support on depression; **(B)** added the reciprocal effects between teacher support and peer support on A; **(C)** examined the effect of depression on teacher support and peer support; **(D)** added the reciprocal effects between teacher support and peer support on C; **(E)** combined A and C; **(F)** combined B and D. TS, teacher support; PS, peer support; T1, Time 1; T2, Time2.

We first tested the effects of teacher support and peer support on depression in Model 1 (**Figure 1A**). Then, we examined the relationship of teacher support at Time 1 to peer support at Time 2 and of peer support at Time 1 to teacher support at Time 2 over Model 1 (Model 2, see **Figure 1B**). Model 3 examined the effect of depression on teacher support and peer support (**Figure 1C**). Model 4 tested the influence of teacher support at Time 1 on peer support at Time 2 and of peer support at Time 1 on teacher support at Time 2 over Model 3 (**Figure 1D**). Model 5 integrated Model 1 and Model 3 (**Figure 1E**), while Model 6 incorporated Model 2 and Model 4 to examine the reciprocal causation between teacher support, peer support, and depression (**Figure 1F**). The participants were grouped into boys and girls when we compared the models.

As seen from **Table 3**, the results from Model 2, Model 4 and Model 6 indicated a good fit to the data. The difference between Chi squares was not significant for Model 6 compared to Model 4 (Δ*χ*^2^ (8) = 2.17, *p* > 0.05). The fit statistics in Model 4 were better than those in Model 2. According to the rule of optimization and simplification, these results suggest that Model 4 is the model that best fits our data.

**Table 3:** Model fit statistics comparing fit of hypothesized model (Model 1 and Model 3) with alternative models.

	χ^2^ (*df*)	χ^2^/*df*	CFI	TLI	RMSEA	*P*
Model 1 (T1, TS, and T1 PS to T2 depression)	96.524 (8)	12.07	0.975	0.924	0.095	<0.01
Model 2 (Model 1 + T1 TS to T2 PS + T1PS to T2, TS)	9.807 (4)	2.45	0.998	0.990	0.034	<0.05
Model 3 (T1 depression to T2 TS and PS)	91.658 (8)	11.46	0.976	0.928	0.092	<0.01
Model 4 (Model 3 + T1 and TS to T2, PS + T1, PS to T2 TS)	5.408 (4)	1.35	1.000	0.998	0.017	>0.05
Model 5 (Model 1 + Model 3)	84.578 (4)	21.14	0.977	0.862	0.128	<0.01
Model 6 (Model 2 + Model 4)	7.574 (12)	0.63	1.000	1.003	0.000	>0.05

*Note. TS = teacher support PS = peer support, T1 = Time 1, T2 = Time2.*

According to these findings, it was assumed that the better fitting causal ordering for these data was from depression to social support. In fact, there was a non-significant difference toward the association of depression with teacher support and peer support between females and males (Wald test of parameter constraints value in Model 4 is 6.987, *df* = 7, *p* > 0. 05). After controlling for age and gender, we computed Model 4 again. The results are as follows: χ^2^ / *df* = 2.838, *p* > 0.05, CFI = 1.00, TLI = 0.99, RMSEA = 0.027, 90 percent CI = 0.000 to 0.055.

Bootstrapping technique was used to assess the significance of the whole model (Shrout and Bolger, 2002; Zhang, 2016). Using a series of random sampling with replacement, 1000 bootstrap samples were generated from the data set (*N* = 2453). The direct paths in the final model were evaluated using maximum likelihood estimation (Zhang, 2016). The results from the bootstrapping analysis are presented in **Table 4**.

**Table 4:** Direct paths and 95% confidence intervals for the final model.

Model pathways	β	*P*	95%CI	
			Lower bounds	Upper bounds
1. T1 TS-T2 TS	0.526	<0.01	0.488	0.563
2. T1 PS-T2 PS	0.553	<0.01	0.517	0.588
3. T1 depression-T2 depression	0.631	<0.01	0.601	0.663
4. T1 depression-T2 TS	-0.011	>0.05	-0.035	0.010
5. T1 depression-T2 PS	-0.033	<0.01	-0.053	-0.016
6. T1 TS-T2 PS	0.081	<0.01	0.049	0.110
7. T1 PS-T2 TS	0.162	<0.01	0.119	0.204

*Note. Path values are standardized regression coefficients.*

Based on the results, depression at Time 1 had a significant effect on peer support at Time 2 (β = −0.03, *p* < 0.01). However, we found a non-significant path from depression in Time 1 to teacher support at Time 2. In addition, teacher support and peer support were closely linked and mutually affected (β_T1__TS-T2_ _PS_ = 0.081, *p* < 0.01; β_T1__TS-T2_ _PS_ = 0.162, *p* < 0.01). From the 95% CI, the effect of peer support at Time 1 on teacher support at Time 2 was significantly larger than the effect of teacher support on peer support. These results provide partial support for our hypothesis.

On average, the set of predictors accounted for 29.8% of the variance in teacher support at Time 2 and 39.9% of the variance in peer support at Time 2. Depression at Time 1 accounted for 36.4% of the variance in depression at Time 2.

## Discussion

This longitudinal study examined the causal relations among teacher support, peer support and depression among adolescents in China. The present study aimed to understand the debate over the relations between social support and depression. The findings of this study demonstrated that depressive symptoms resulted in a decrease in perceived support from peers but not from teachers. Teacher support and peer support were closely linked with each other and mutually influential.

Consistent with prior studies (e.g., Stice et al., 2004), the result of this study indicated that depression influenced peer support significantly and negatively. Adolescents who are less depressed obtain peer support more easily. The current findings may be explained by interpersonal theories asserting that depression promotes support erosion. There are two possible explanations for the interpersonal theories of depression (Daley and Hammen, 2002; Rudolph et al., 2008). One possible explanation is that depressed individuals may generate negative and rejecting responses through premature self-disclosure and self-criticism, which make others feel uncomfortable and result in avoiding the depressed person. A second plausible explanation is that depressed adolescents lack necessary social ability and tend to avoid social situations, which may cause lower perceived support by the adolescent.

However, these support erosion effects may be pronounced for peer support but not for adult support, such as for teacher support in this study or parental support in the study of Stice et al. (2004, 2011). These less pronounced effects may be because parents and teachers feel a sense of responsibility to provide help and care for the depressed adolescents, which is not evoked for peers (Stice et al., 2011). Regarding gender, there was no significant difference in the relation between social support and depression when examined by gender, which indicated that decreased depression symptoms in both males and females promoted their perceived peer support. Our research suggests that decreasing depression may be a worthy target of intervention to influence peer support.

We were surprised to find that peer support and teacher support did not affect adolescent depression in our study, which did not support the social causation model. These findings were contrary to those reporting that social support was considered to be protective against depression (e.g., Reddy et al., 2003; Rueger et al., 2016). Low levels of peer support were not linked to subsequent depressive symptoms. A possible reason for these conflicting findings might be that previous attempts to study the impact of social support likely inflated the independently predictive role of social support without adjusting for possible confounders (Dingfelder et al., 2010). Marroquín (2011) suggested that although perceived social support seemed clearly linked to depression, the exact nature of this relationship remains an open question. Such findings were consistent with previous research (Vaughan et al., 2010; Auerbach et al., 2011), which indicated that peer support had little or no influence on subsequent depressive symptoms.

Our results did not support the negative spiral relations between social support and depression. The results of previous studies were inconsistent. Hansell and Damour (2005) suggested that people were more prone to depression when perceiving lower levels of social support and when people were depressed, it was impossible for them to seek or maintain relationships. Thus, there was a negative spiral between social support and depression (Cooley et al., 2010). For example, perceived parental support influenced children’s trajectories of depression during adolescence, which in turn affected their perceived parental support in young adulthood (Needham, 2008). However, among 11–16 years old, parental support was not associated with adolescents’ depression 2 years later, and depression did not cause changes in parental support over time (Young et al., 2005).

Another notable result in this study is that although teacher support is a precursor to peer support, peer support also affects teacher support. Our finding indicated that teacher support and peer support were mutually influential is consistent with Bronfenbrenner’s (1977) ecological system perspective. According to the theory, the social systems can not only have a direct effect on the individual child but also interact with one another. There may be some certain interactions that can occur between the support systems. Previous studies have indicated that students who perceive higher support from classmates often have higher support from teacher as well (Ansong et al., 2017), and students who seek support from their peers tend to seek help from their teachers (Hamilton, 2013). It could be that some qualities within a student not only attract peers to support him or her but also urge teachers’ help.

The present study is limited in several respects. Specifically, the variables used in the current study are subjective, self-report measures, which increase the method bias in the measurement (Ansong et al., 2017). Second, only two waves were included in this study, which resulted in our results being limited to two waves of data. This half-year gap between the two waves may have made our estimates of the relations between social support and depression rather conservative. Third, our sample focuses on adolescent students. When participants do not fit this demographic profile, conclusions need to be made with caution (Wentzel et al., 2010). These sampling issues require further careful consideration in future studies in this area. A fourth limitation is that the present study only includes two sources of social support (teacher support and peer support). Parental support is also an important source of social support for adolescents; however, it is beyond the scope of the current study. Future research should benefit from the replication of the findings through more waves of longitudinal data and more social support sources (e.g., parents, teachers, classmates, and peers).

Despite these limitations, we are confident that the findings of this study indicate that depression erodes social support, which provides additional support for the interpersonal theories of depression. However, the effect is limited to peer support, but not to teacher support. The current work contributes to the debate on the relation between social support and depression. Clearly, the association between social support and depression presents a much more complex area of research than researchers have thought.

## Ethics Statement

This study was carried out in accordance with the recommendations of Ethical Conduct in Human Research by the Ethical Review Boards of Collaborative Innovation Center of Assessment toward Basic Education Quality, Beijing Normal University, with written informed consent from all subjects. All subjects gave written informed consent in accordance with the Declaration of Helsinki. The protocol was approved by the Ethical Review Boards of Collaborative Innovation Center of Assessment toward Basic Education Quality, Beijing Normal University.

## Author Contributions

PR, YZ, and RZ contributed to the writing of the manuscript and data analysis. XQ conducted the main statistical analysis and edited the manuscript. YZ collected the study data and conducted the most part of the preliminary data analysis. PR, XQ, YZ, and RZ contributed to this paper and approved it for publication.

## FUNDING

This study was supported by the National Social Science Foundation of China (Project No. 17BSH100) and Henan Provincial Philosophical and Social Science Foundation of China (No. 2015BJY015).

## Conflict of Interest Statement

The authors declare that the research was conducted in the absence of any commercial or financial relationships that could be construed as a potential conflict of interest.
